# Methylation of *DLEC1* Promoter Is a Predictor for Recurrence in Chinese Patients with Gastric Cancer

**DOI:** 10.1155/2014/804023

**Published:** 2014-12-09

**Authors:** Xiaobing Ye, Gang Feng, Nanlin Jiao, Chun Pu, Guohai Zhao, Guoping Sun

**Affiliations:** ^1^Department of Medical Oncology, The First Affiliated Hospital of Anhui Medical University, Hefei, Anhui 230001, China; ^2^Department of Medical Oncology, The First Affiliated Hospital of Wannan Medical College, Wuhu, Anhui 241001, China; ^3^Clinical Genetics Laboratory, The First Affiliated Hospital of Wannan Medical College, Wuhu, Anhui 241001, China; ^4^Department of Pathology, The First Affiliated Hospital of Wannan Medical College, Wuhu, Anhui 241001, China; ^5^Department of Surgery, The First Affiliated Hospital of Wannan Medical College, Wuhu, Anhui 241001, China

## Abstract

*Purpose*. To investigate promoter methylation in the deleted in lung and esophageal cancer 1 (*DLEC1*) gene in Chinese patients with gastric cancer. *Methods*. A total of 227 patients with gastric cancer were enrolled. The methylations of the promoter regions of *DLEC1* and ACTB were determined using quantitative methylation-specific PCR. The *DLEC1* methylation was compared to the clinicopathological variables of gastric cancer. *Results*. *DLEC1* methylation was not associated with the clinicopathological variables of gastric cancer. Patients with *DLEC1*-hypermethylated gastric cancer had significantly higher recurrence rate than those with *DLEC1*-hypomethylated gastric cancer (*P* = 0.025; hazard ratio = 2.43). *Conclusions*. Methylation of DELC1 promoter may be a valuable predictor for recurrence in Chinese patients with gastric cancer.

## 1. Introduction

Gastric cancer is one of the most common malignancies and remains an important cause of mortality worldwide, especially in Asia [1, 2]. The combination of surgical resection and adjuvant chemo- or radiotherapy has provided a significant improvement for the survival of patients with localized gastric cancer [3]. However, about 80% of the patients die within a short period of time from recurrence after curative surgery [4]. Therefore, early detection of recurrence is important for evaluating the treatment outcome and choosing the most effective management in patients with gastric cancer.

Contrast enhanced computed tomography (CT) is the most frequently used imaging modality for the detection of gastric cancer recurrence [5]. However, CT cannot reflect the presence and viability of cancer recurrence precisely because its diagnostic ability is dependent only on morphological changes of the involved organs and distorted anatomical structures [6]. Recently, integrated positron emission tomography (PET) with CT (PET-CT) for detection of gastric cancer recurrence after surgical resection has been reported [7–9].

Compared to more expensive imaging methods, analyses of tumor biomarkers have no risk of radiation exposure, are easily available, and are more cost effective. Accordingly, studies on tumor molecular markers in prognosis of gastric cancer are relevant. Many studies indicate that promoter CpG island hypermethylation is closely associated with inactivation of tumor suppressor genes in human cancers. Furthermore, all types of human cancer display promoter CpG island hypermethylation, although there are variations in the prevalence of CpG island hypermethylation among tumor types [10, 11]. The stomach is one of the organs where aberrant CpG island hypermethylation occurs frequently during cancer development [12]. Many genes have been characterized to be inactivated by hypermethylation of their promoter CpG islands in gastric cancer [13].

Deleted in lung and esophageal cancer 1 (*DLEC1*) is a tumor-suppressor gene which suppresses tumor growth or reduces the invasiveness of cancer cells and promoter hypermethylation has been shown to be responsible for the silencing of* DLEC1* in ovarian cancer and nasopharyngeal carcinoma [14]. Furthermore, promoter hypermethylation of* DLEC1* has also been found in gastric cancer [15]. Ying et al. demonstrated that* DLEC1* was downregulated or silenced in most gastric cell lines due to promoter methylation, whereas it was broadly expressed in normal stomach tissues [16].

The purpose of our study is to investigate the relationship of* DLEC1* methylation with clinicopathologic variables and determine whether* DLEC1* methylation has any prognostic significance in patients with gastric cancer.

## 2. Materials and Methods

### 2.1. Patients

The study group included gastric cancer patients who had undergone radical surgical resection (D2) from Jun 2008 to Jun 2010. All tissues were fixed in 10% neutralized formalin, embedded in paraffin, cut into 4 *μ*m sections, and stained with hematoxylin and eosin (H&E) in order to confirm the histological diagnosis and microscopic characteristics of the specimens. The staging for each gastric cancer was evaluated according to the Union for International Cancer Control system, which indicates the extent of tumor spread [17]. Histological architecture was defined using the Lauren classification [18]. The tumor size, depth of invasion, lymphatic and venous invasion, and lymph node metastasis of tumors were also determined.

No patients were treated with chemotherapy, radiotherapy, and adjuvant treatment prior to surgery. All patients except stage I patients were also treated with standard adjuvant chemotherapy of modified FOLFOX6 regimen.

Follow-up information about the postoperative clinical course of patients was available from outpatient medical records or telephone calls. Recurrence-free survival (RFS) was defined at the time of surgery to tumor recurrence. The end date of the follow-up study for conducting the analysis was Jun 2014. The study protocol was approved by the First Affiliated Hospital of Wanan Medical College and the First Affiliated Hospital of Anhui Medical University.

### 2.2. DNA Extraction and Bisulfite Treatment

Ten sections of 10 mm thickness of paraffin-embedded tissues were used for DNA extraction. The paraffin was removed from the tissue by rinsing in xylene and genomic DNA was isolated using a QIAamp tissue kit (Qiagen, Valencia, CA, USA). DNAs were stored at −80°C before analysis. DNA (1 *μ*g) was treated with bisulfite to convert unmethylated cytosines to uracils using the EZ DNA Methylation-Gold Kit (Zymo Research Corporation, Irvine CA, USA) according to the manufacturer's protocol. After treatment, DNAs were stored at −80°C until being used.

### 2.3. Quantitative Methylation-Specific PCR

The methylations of promoter were determined using quantitative methylation-specific PCR. The primers and probe for* DLEC1* were 5′-TTT CGT TGC GTA TTT AAG ATA TTT C-3′, 5′-CGT AAC GCT CAT TCT CGC TAC C-3′, and 6-FAM-5′-TAA TCA AAC TTA CGC TCA CTT CGT CGC CG-3′-6-TAMRA. The primers and probe for ACTB were 5′-TGG TGA TGG AGG AGG TTT AGT AAG T-3′, 5′-AAC CAA TAA AAC CTA CTC CTC CCT TAA-3′, and 6-FAM-5′-ACC ACC ACC CAA CAC ACA ATA ACA AAC ACA-3′-6-TAMRA. Amplification reactions were carried out in triplicate in a final volume of 20 *μ*L that contained 3 *μ*L of bisulfite-modified DNA; 600 nM concentrations of forward and reverse primers; 200 nM probe; 0.6 U of platinum Taq polymerase (Invitrogen, Frederick, MD); 200 mM concentrations each of dATP, dCTP, dGTP, and dTTP; and 6.7 mM MgCl_2_. Amplifications were carried out using the following program: 95°C for 3 minutes, followed by 40 cycles at 95°C for 15 seconds, and 60°C for 1 minute. Amplification reactions were carried out in 384-well plates in Roche LightCycler 480-II (Roche Applied Science) and were analyzed by LightCycler 480 software (version 1.3). Each plate included patient DNA samples, positive (in vitro methylated leukocyte DNA) and negative (normal leukocyte DNA) controls, and water blanks. A standard curve was generated using serial dilutions of CpGenome Universal Methylated DNA (Chemicon, Temecula, CA).* DLEC1* methylation was defined as a ratio of methylation specific PCR-amplified* DLEC1* to ACTB and then multiplied by 100 for easier tabulation.

### 2.4. Statistical Analyses

*DLEC1* methylations were expressed as mean ± SD. The associations between* DLEC1* methylation and the clinicopathological variables were assessed by Mann-Whitney* U* test. Receiver operator curves (ROC) were used to compare the ability to identify patients with recurrence by* DLEC1* methylation. RFS was generated using Kaplan-Meier estimates, and the difference between curves was evaluated with the Log-rank test. Hazard ratios (HRs) and 95% confidence intervals (CIs) computed from multivariate analysis were used to investigate the relationship between RFS and variables. Differences were considered significant at a level of *P* < 0.05. All statistical analyses were performed using the SPSS 13.0 statistical package.

## 3. Results

A total of 227 patients with gastric cancer were enrolled into the study. There were 157 males and 70 females with age of 63.15 ± 12.02 years (range 35–86). The associations between* DLEC1* methylation and clinicopathological variables were shown in Table 1.* DLEC1* methylation was not associated with age and gender (*P* = 0.392, *P* = 0.421). In addition,* DLEC1* methylation did not correlate with tumor size (*P* = 0.243), depth of invasion (*P* = 0.066), lymphatic invasion (*P* = 0.102), venous invasion (*P* = 0.074), TNM staging (*P* = 0.063), Lauren classification (*P* = 0.050), and lymph node metastasis (*P* = 0.089).

**Figure 1:**
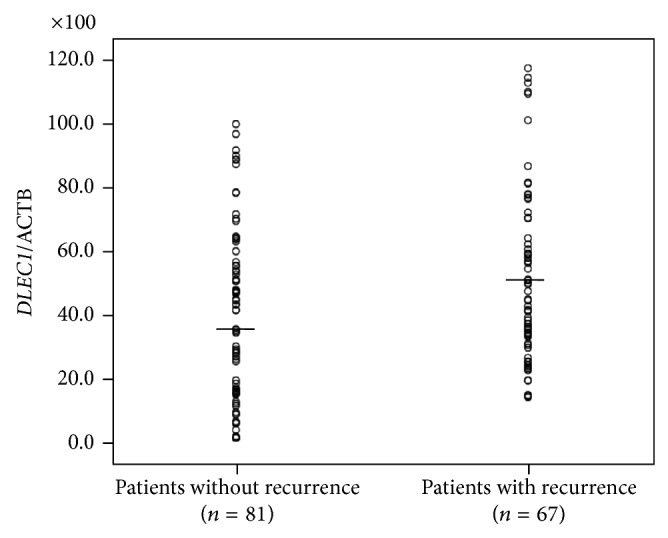
Scatter plots showing methylation levels of* DLEC1* in gastric tumor separated by recurrence. Calculation of* DLEC1* to ACTB ratio was based on the fluorescence emission intensity values for both genes obtained by quantitative methylation-specific PCR analysis. The obtained ratios were multiplied by 100 for easier tabulation.

**Figure 2:**
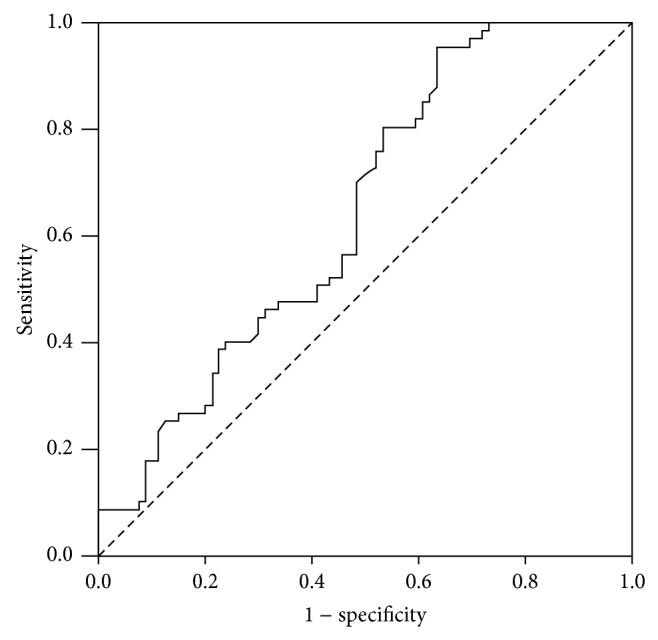
Receiver operating characteristic (ROC) curve of* DLEC1* methylation in predicting recurrence in patient without lymph node metastasis after surgery. The area under the curve (AUC) was 0.648 (0.560–0.736). The best cutoff value was 35.10 (sensitivity, 70.1%; specificity, 51.9%).

Among 148 patients without lymph node metastasis, 67 (45.3%) patients were found to have a recurrence after surgery.  Table 2 described the sensitivity and specificity of clinicopathological factors for recurrence.* DLEC1* methylation was significantly higher in patients with recurrence, as compared with that in patients without recurrence (*P* = 0.012, Figure 1). ROC analyses of* DLEC1* methylation in patients with and without recurrence are shown in Figure 2. In this study population, the best cut-off point for* DLEC1* methylation was 35.10.* DLEC1* methylation of 35.10 demonstrated a sensitivity and specificity of 70.1% and 51.9%, respectively, for recurrence (ROC AUC = 0.648; 95% CI, 0.560–0.736).

**Figure 3:**
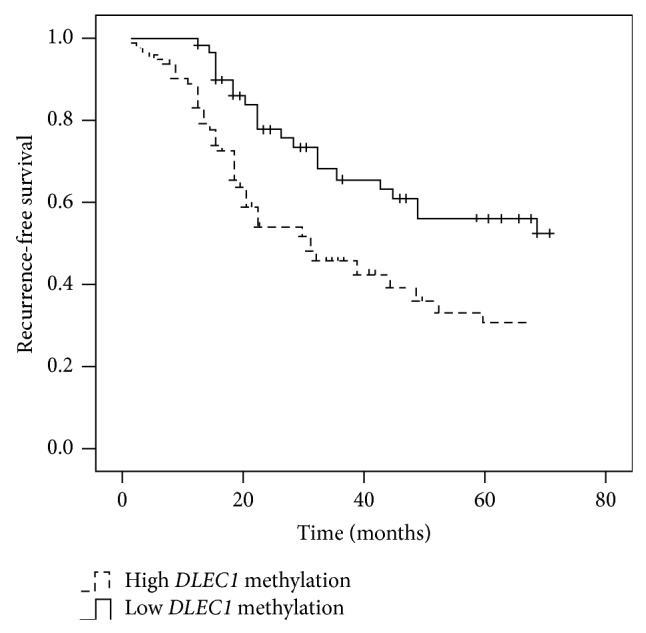
The recurrence-free survival curves based on* DLEC1* methylation. Patients with high* DLEC1* methylation had a significantly higher recurrence rate than those with low* DLEC1* methylation (*P* = 0.025).

Figure 3 showed that Kaplan-Meier analysis of RFS based on* DLEC1* methylation using 35.10 as the optimal threshold.* DLEC1* methylation was associated with RFS in the evaluated cohort (*P* = 0.028). A multivariate Cox proportional hazards model using variables associated with RFS in our study indicated that depth of invasion, lymphatic invasion, venous invasion, TNM staging, Lauren classification, and lymph node metastasis (*P* < 0.05), but not age, gender, or tumor size (*P* > 0.05), were independent predicted factors for recurrence in gastric cancer. Although the impact of* DLEC1* methylation on RFS was less evident than depth of invasion, lymphatic invasion, venous invasion, Lauren classification, and lymph node metastasis, the risk of recurrence in patients with higher* DLEC1* methylation was still 2.43 times higher than those with lower* DLEC1* methylation (*P* = 0.025) (Table 3).

## 4. Discussion

CpG islands are DNA segments, at least 0.5 kb in size, rich in G:C and CpG content, and often located in the promoter or 50-exon sequences of genes. Promoter CpG islands have traditionally been thought to be unmethylated in normal cells. Although the cause is unclear, promoter CpG island hypermethylation can occur in association with cancer development or aging. Promoter CpG island hypermethylation is biologically important for gene function and thought to act as an alternative to genetic change for inactivation of tumor suppressor genes in human gastric cancer [15].

The* DLEC1* gene firstly is deleted in lung cancer and located in the 3p22.3 region, which has been identified as one of the common deleted regions in lung cancer [19].* DLEC1* gene encodes a protein which has no significant homology to known proteins or domains and the function of which remains unknown [20]. Functional analyses strongly suggest that* DLEC1* is a tumor suppressor gene [21]. Previous research demonstrated the loss of* DLEC1* expression in ovarian cancer and the suppression of ovarian cancer cell growth by* DLEC1* reexpression. The loss of* DLEC1* expression in ovarian cancer is related to promoter hypermethylation and histone hypoacetylation but not to loss of chromosome 3p22.3 [14]. Many studies showed that methylation of the* DLEC1* gene correlates with poor prognosis in lung cancer and ovarian cancer [22–24].

In this study, we determined methylation of* DLEC1* promoter by quantitative methylation-specific PCR and demonstrated that* DLEC1* promoter was hypermethylated in Chinese gastric cancer patients. However, we did not find any correlations between* DLEC1* methylation and clinicopathological variables in Chinese gastric cancer patients. Our investigation was similar to other previous studies [16, 23].

Tumour-specific promoter methylation can serve as a biomarker for prognosis of tumor [25, 26]. We had found that* DLEC1* methylation was significantly higher in patients with recurrence, as compared with that in patients without recurrence among patients without lymph node metastasis. ROC analyses demonstrated that* DLEC1* methylation had sensitivity and specificity of 70.1% and 51.9%, respectively, for recurrence (ROC AUC = 0.648; 95% CI, 0.560–0.736). In our study, a multivariate Cox proportional hazards model indicated that* DLEC1* methylation was an independent risk factor for recurrence in gastric cancer. Thus, methylation of* DLEC1* may be a valuable indicator for recurrence in gastric cancer.

There are some limitations in our study. First, our study population was relatively small and from a single center. Second, we had not measured* DLEC1* RNA expression and* DLEC1* protein expression in tissues of gastric cancer. However, it would be worthy further exploring the possible use of* DLEC1* methylation as a predictor for recurrence in gastric cancer. The multicentric and large-scale prospective validation studies are required in order to confirm our present findings.

## 5. Conclusions

In conclusion, methylation of DELC1 promotermay be a valuable predictor for recurrence in gastric cancer patients.

## Figures and Tables

**Table 1:** Correlation between clinicopathological variables and *DLEC1* methylation.

Clinicopathological variables	Number	*DLEC1* methylation	*P* value
Age (years)			0.392
≤65	117	49.27 ± 28.98	
>65	110	48.54 ± 36.04	
Gender			0.421
Male	157	48.90 ± 29.73	
Female	70	48.77 ± 38.29	
Tumor size (cm)			0.243
<4	118	46.56 ± 30.78	
≥4	109	51.44 ± 32.06	
Depth of invasion			0.066
Tis-1	116	45.05 ± 31.05	
T2-4	111	52.94 ± 33.43	
Lymphatic invasion			0.102
Positive	82	53.88 ± 37.61	
Negative	145	46.09 ± 28.02	
Venous invasion			0.074
Positive	33	58.89 ± 35.19	
Negative	194	47.21 ± 31.57	
UICC TNM staging			0.063
0-I	41	42.01 ± 24.49	
II–IV	186	50.43 ± 33.21	
Lauren classification			0.050
Intestinal type	122	44.12 ± 29.50	
Diffuse type	105	54.48 ± 35.04	
Lymph node metastasis			0.089
Positive	79	43.98 ± 27.94	
Negative	148	51.54 ± 38.22	

**Table 2:** The sensitivity and specificity of clinicopathological factors for recurrence.

Factors		Recurrence	Sensitivity	Specificity	PPV	NPV
Tumor size (cm)						
<4	85	38	43.3%	58.0%	46.0%	55.3%
≥4	63	29				
Depth of invasion						
Tis-1	102	27	59.7%	92.5%	86.9%	73.5%
T2-4	46	40				
Lymphatic invasion						
Positive	18	16	23.9%	97.5%	88.9%	60.8%
Negative	130	51				
Venous invasion						
Positive	9	7	10.4%	97.5%	77.8%	56.8%
Negative	139	60				
TNM staging						
0-I	41	13	80.6%	34.7%	50.5%	68.3%
II–IV	107	54				
Lauren classification						
Intestinal type	82	25	62.7%	70.4%	63.6%	69.5%
Diffuse type	66	42				

**Table 3:** Multivariate analysis of recurrence-free survival in gastric cancer according to clinicopathological variables and *DLEC1* methylation.

Clinicopathological variables	HR	95% CI	*P* value
Age (≤65 versus >65)	1.12	0.91–2.04	0.073
Gender (male versus > female)	1.05	0.70–1.21	0.103
Tumor size (<4 versus ≥4)	1.58	1.16–2.26	0.058
Depth of invasion (Tis-1 versus T2-4)	3.81	1.68–6.85	0.008
Lymphatic invasion (+ versus −)	4.54	2.06–7.36	0.001
Venous invasion (+ versus −)	2.86	1.43–5.01	0.017
TNM staging (0-I versus II–IV)	2.32	1.26–4.83	0.036
Lauren classification (intestinal versus diffuse type)	3.66	1.37–8.94	0.009
Lymph node metastasis (+ versus −)	3.92	1.78–7.15	0.002
*DLEC1* methylation (>35.10 versus ≤35.10)	2.43	1.38–5.07	0.025
